# Wealth inhomogeneity applied to crash rate theory

**DOI:** 10.1016/j.heliyon.2015.e00041

**Published:** 2015-11-24

**Authors:** Robert L. Shuler

**Affiliations:** NASA Johnson Space Center, Houston, TX 77058, United States

**Keywords:** Economics, Political economics, Monetary economics, Methodology in economics, Social policy, Social issues, Public welfare, Methodology of social sciences, Risk analysis

## Abstract

A crash rate theory based on corporate economic utility maximization is applied to individual behavior in U.S. and German motorway death rates, by using wealth inhomogeneity data in ten-percentile bins to account for variations of utility maximization in the population. Germany and the U.S. have similar median wealth figures, a well-known indicator of accident risk, but different motorway death rates. It is found that inhomogeneity in roughly the 10^th^ to 30^th^ percentile, not revealed by popular measures such as the Gini index which focus on differences at the higher percentiles, provides a satisfactory explanation of the data. The inhomogeneity analysis reduces data disparity from a factor of 2.88 to 1.75 as compared with median wealth assumed homogeneity, and further to 1.09 with average wealth assumed homogeneity. The first reduction from 2.88 to 1.75 is attributable to inequality at lower percentiles and suggests it may be as important in indicating socioeconomic risk as extremes in the upper percentile ranges, and that therefore the U.S. socioeconomic risk may be higher than generally realized.

## 1 Introduction

Systematic study of risk through enumeration of outcomes and likelihoods is as old as writing and civilization [1]. The oldest approach is *risk analysis*, based on a statistical and engineering hypothesis about the probability or reliability of component events or parts, and still dominates engineering and policy approaches today. By the 17^th^ century de Meer, Pascal, Fermat, Bernoulli, de Moivre, Bayes and others developed tools of *risk management* to address risks which could not be avoided, using tools such as insurance, futures and derivatives [2]. Markowitz portfolio theory [3] and its variations are in this vein, and risk management has become a dominant approach in business and finance, though the term is also used in engineering to mean merely the management of various components of the older risk analysis approach.

In 1975 the idea of *risk compensation* was introduced by Peltzman [4] and others who argued that consumers of products to which safety features had been added might maximize some other utility, causing the improvement of safety in usage to be less than predicted by the raw engineering data. An understanding began to emerge that risk was not always the simple sum of its components. This was used to explain seat belt and airbag crash data. Wilde in 1982 suggested the name *risk homeostasis* to emphasize the idea of a feedback loop in which the consumer tended to maintain a comfortable level of target risk, a risk thermostat [5]. While engineers and policy makers chaff at what they take as a limiting view of their ability to control risk by direct methods, data continue to pile up supporting the validity of this personal psychological feedback model [6], [7].

By 1987 Slovic questioned whether humans – the general public and participants in risk activities, not necessarily specialists – accurately perceive new or technologically complex risks, with which they have not had time to come into evolutionary equilibrium, in other words for which instinct cannot be relied upon and analysis is required [8]. Such a misperception would, if large enough, defeat an instinctive calculus of risk compensation or homeostasis, but could be addressed through education, and through direct mitigation which we have already attributed to engineers and policy makers. This leaves a great amount of room for improvement through analysis of individual components of risk and infrastructure or policy actions. Eventually this led to risk density diagrams from which can be identified the most productive candidates for risk reduction (for example dangerous curves on rural roads) and corresponding policies to anticipate discrepancies between actual and perceived risk (for example guidelines for maximum rate of change of curvature on roads of various categories) [9], [10]. But when funds to implement this approach are available, as in wealthy countries such as Germany and the U.S., and puzzles remain in the data, then one must look to other explanations.

Early in the 2000's a view of risk was developed from the fields of project risk management, corporate economic behavior (as opposed to individual psychological behavior), and the verification and failure rate prediction of complex systems such as computer software, aircraft or spacecraft [11]. Though based on economic principles, feedback loops, and corporate behavior, there were some similarities in effect to the psychological risk compensation theories for individuals. In this approach, which could be called crash rate theory, operational crash rate *R_o_* (aggregated risk loss rate) is related to five parameters expressed as a profit-maximizing feedback system: the value of the service, system or added features under consideration *V_f_*, the cost of providing or manufacturing the service or features *M*, the cost of a crash *C_c_*, the cost of developing and testing the service or features *C_d_*, and the defect ratio *D* (ratio of defects in delivered products or services to those found during inspection or testing). These parameters are incrementally valid for reasonable sized changes to processes that may be modeled linearly. The static equilibrium solution in a competitive environment can be written as a crash rate equation:

(1) $$R_{o}\;\;\approx \frac{V_{f}\;-\;M\;}{C_{c}\;+\;C_{d}/D}$$

Although crash rate theory is derived from economic assumptions, and relies upon economic competition for its ultimate enforcement, not psychological preferences, the statistics supporting risk homeostasis were used to justify the general argument. Conversely, risk compensation or risk homeostasis originally used the language of “maximizing utility,” an economic term, in its justification. As the scope envisioned for applicability of the crash rate equation gradually expanded to include non-profit and government projects, it began to be suspected it might apply to large groups of people, or society generally [12].

At some point researchers from each orientation were discussing the similarities, and the one with a risk homeostasis background asked the one with the project and economic background, “Can you explain the U.S. motorway death rate?” For example, the U.S. death rate per 100,000 persons per year is 11.3, whereas Germany, with some unlimited speed roads and one of the higher death rates in Europe, and similar average and even median wealth to the U.S., has a motorway death rate of only 4.0 per 100,000. Given that an overall inverse correlation between wealth of countries and their motorway death rates is well known, and the apparent similarity of the U.S. and Germany, their disparity in death rates constitutes a puzzle. A solution to this puzzle might provide new insights.

## 2 Methods

The first point of methodology is in selection of units for the numerator and denominator for the rates to be compared. The crash rate theory can handle any units, but one must select based on relevance and data availability. It is also desirable to select data which is not otherwise well understood, so as to test the usefulness of our new methodology combining crash rate theory and inhomogeneity.

It is likely that any diligently executed and self-consistent crash rate methodology can, with sufficient data and care, explain any given set of crash rates. For example, it is possible to look at road conditions, traffic levels, safety enforcement, driver education policy, road utilization and so forth, and come to equally valid explanations. The art of explaining merely converts one set of parameters to another, assumed to be independent. But in reality, all these are sociological parameters and are all interdependent. If one wishes to examine whether inhomogeneity has any role, then one uses a methodology that has the possibility of including this parameter. That does not invalidate other established and credible methodologies. It is even possible to make the German-U.S. anomaly seem to “go away” by choosing to measure deaths per mile. This introduces new parameters, such as differences in utilization of the roads, population density, and the value of driving a mile (or kilometer) in each country. The choice of methodology in this study is made to investigate the relevance of the crash rate equation and an associated inhomogeneity methodology, and not to detract from the relevance of other approaches.

While some in the safety community use deaths per mile of roadway, this can be biased by under-utilized roads. Transportation specialists, concerned with the engineering of safe transportation, often use deaths per mile driven. However, miles driven on under-utilized roads might be safer than miles driven in heavy traffic. In this measure, Germany appears safer than the U.S., but the U.S. has built thousands of miles of interstate highways through desert and near-desert regions which carry little traffic. One original purpose of the interstate system was military mobility, not as a response to ordinary traffic demand [13]. Such a road-building strategy negates any usefulness of per mile data. Even per mile driven data is biased by the exceptionally light traffic on these roads.

Consider setting up the crash rate equation for the per mile driven case, and for simplicity consider just the ratio *V_f_*/*C_c_*. One would need the value of driving per mile driven to yield an *R_o_* corresponding to crashes per mile driven. Is the value of a trip to the nearest vacation resort or large commercial city dependent on the distance to it? The value is more likely dependent on the fact that it is the nearest one, and the wealth of the person taking vacation or the value of the commercial activity. Double the distance and the value per mile simply drops in half. Therefore while per mile data is quite useful to road building engineers, who are not concerned with the value of driving the mile, it is less important to an economic model of crash rate.

In the per capita per year case, the U.S. appears 2.85 times more dangerous than Germany. The number of one's friends or family members killed is a palpable influence on behavior that is likely to be felt similarly in any developed country in which violent and unexpected death is relatively rare. This way of viewing the data provides the sort of “puzzle” needed to validate the methodology herein. The U.S. and Germany are comparable in wealth and in diversity of destinations, but as the U.S. is much larger, destinations can be much farther apart. If the value of the destinations is similar, then the value per mile of travel in the U.S. may be lower. If this hypothesis is even approximately true, then the puzzle is a real one.

Next consider the cost of a fatal crash *C_c_* to the victim. The victim loses the use of the rest of his or her life, and whatever resources the victim had. It is possible to give meaning to the other terms as well. For example, in the U.S. with less public transportation, the value of driving *V_f_* may be higher. In Finland and Sweden emphasis is placed on verification, not only by roadside monitoring of speeding and testing for intoxication, but also by increased use of vehicles which require the driver to pass a sobriety test before the vehicle will start [14]. The cost of such measures logically would go into the cost of development and testing *C_d_*, and a defect ratio estimating the rate at which drivers evade roadside monitoring or defeat vehicle sobriety tests could be determined, *D*. The effect of these parameters might be considerable and necessary to construct an explanation of the *per mile* death rates. By using data regarding fatalities *per capita*, and assuming the cost of a crash is of the order of the worth of a person's life, one can assume that for fatal accidents *C_c_*≫*C_d_*/*D*, and neglect *C_d_*/*D*. This would not be the case if non-fatal accidents were considered.

**Fig. 1:**
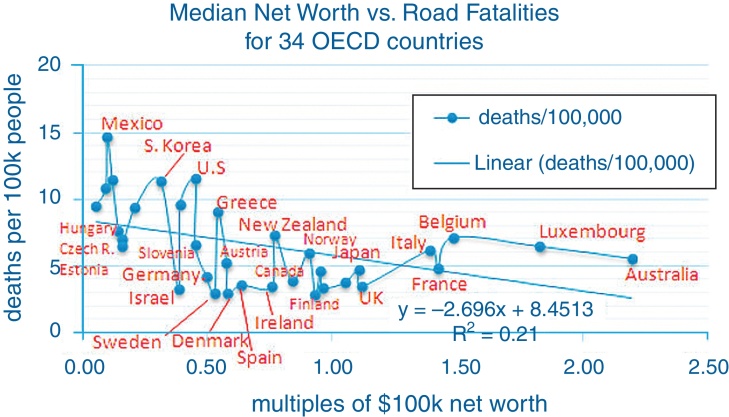
Median net worth vs. road fatalities for 34 OECD (Organization for Economic Cooperation and Development) countries.

A country-specific calibration factor for (*V_f_* – *M*)/*C_c_* is obtained using a weighting factor determined from empirical data for 34 countries in the Organization for Economic Cooperation and Development (OECD) correlating median net worth [15] with motorway fatality rates [16]. For summary data see http://en.wikipedia.org/wiki/List_of_countries_by_wealth_per_adult and http://en.wikipedia.org/wiki/List_of_countries_by_traffic-related_death_rate. Notice in the chart in Fig. 1 that for the two countries chosen, the U.S. and Germany, the median net worth is close to the same, only slightly higher in Germany, but the death rates are nearly at opposite ends of the spectrum. So this is the puzzle to be solved. From the chart, there is a 50% weighting on net worth in the cost of crashes *C_c_* parameter. That is the slope of the computer-generated straight line fit shown.

The median value of net worth (NW) is taken for each country in the chart because it is close to the net worth of a larger number of people for these two countries than the average net worth, so it is likely more representative of behavior in situations where one must take a single number. If relative net worth is normalized so that the median is 1.0 (on a per country basis), then the 50% weighting from the OECD data can be used to compute a net worth impact factor NWIF = 1 + 0.5 (NW − 1). For example, for a relative net worth of 2.0 (twice the median value), this gives *C_c_* ∝ NWIF = 1 + .5 (2−1) = 1.5. All of our calculations are done with net worth normalized to the median for the country to which the calculations apply.

The hypothesis to be tested is that the difference between U.S. and German motorway deaths per capita can be largely explained by differences in the homogeneity of the populations. This might not be an obvious hypothesis to the reader who is aware that Germany has the greatest wealth and income inhomogeneity in Europe as measured by the Gini index. It is the test of the hypothesis which concerns us here. The U.S. has one of the highest inhomogeneity rates in the developed world. The Gini index for pre-tax income is actually higher in Germany, but after adjustments for taxes and distributions, the U.S. Gini index is higher. Based on the measurements used in studies of wealth inhomogeneity today, it is not obvious that it could account for the difference in motorway deaths, and such a result, even if partly true, would be surprising. The present method will look in much more detail at the structure of inhomogeneity than the two-factor Gini index, using instead a 10-factor model.

The next methodological problem was to obtain data on wealth inhomogeneity for the U.S. and Germany which is consistent and comparable. Data from 2013 for net worth were available for both countries, and by sampling and interpolation were accurately binned into ten percentile groupings. These comprise our 10-factor model of wealth inhomogeneity.

**Fig. 2:**
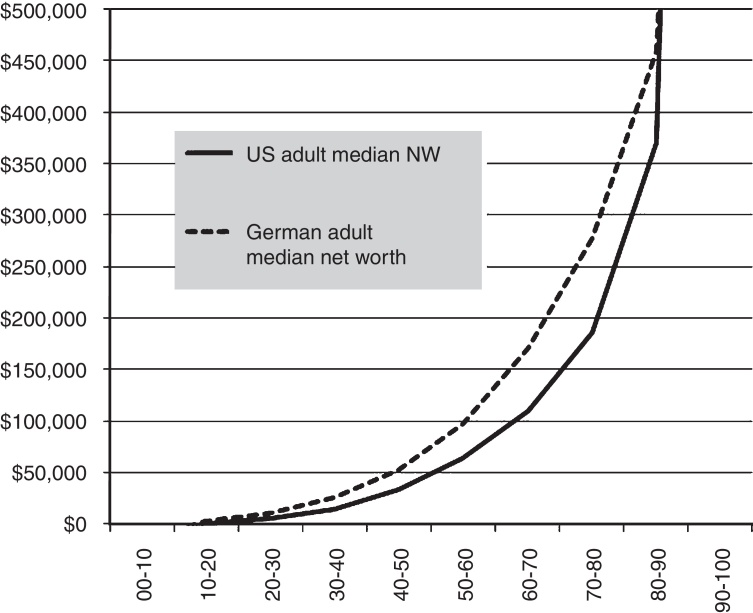
German vs. U.S. median adult net worth percentile bins (USD – U.S. Dollars).

The 0–10 percentile group for both countries had negative net worth and was discarded since the negative numbers do not work well with the ratio method of comparing the data. So actually, only 9 factors are used. There are two reasons why this negative data might not be as important as it first seems. Some with negative net worth may in fact have respectable or even high incomes. And many with negative net worth and no income are unlikely to be drivers. In any case, the Germany 0–10 group was just slightly more negative than the U.S. 0–10 group, and would have raised the inhomogeneity-adjusted rate for Germany contributing to our thesis, so excluding it biases the investigation against our thesis and thus does no harm to our findings. Fig. 2 summarizes the data.

The 90–100 bracket (U.S. ∼$2.5 M and Germany ∼$1.6 M) is not plotted because showing the very high numbers on the same scale spoils the plot. German data are from Bundesbank [17], and U.S. data are from the Federal Reserve Survey of Consumer Finances [18]. The U.S. data were analyzed in part with the aid of the Net Worth Percentile Rank Calculator, Shnugi Personal Finance, http://www.shnugi.com. Household data are in some cases adjusted for comparison with individual adult data.

Using this data, a spreadsheet calculation was used to adjust the median-normalized net worth using the OECD derived net worth impact factor for each percentile bin for each country, giving a relative projected motorway death rate for each percentile bin. These were summed and averaged to give an estimated *relative* death rate per capita, amounting to an inhomogeneity adjustment factor. If the median net worth were universal within the country, this adjustment factor number would have been 1.0. Since it is greater than 1.0 for both the U.S. and Germany, it indicates the degree to which the death rate reflects an inhomogeneous population. For example, an adjustment factor of 2.0 for a particular percentile bin suggests the actual motorway death rate for that group is twice what it *would be* if the country were homogeneous in net worth at the median value. To get any other value, for example the rate one would estimate if everyone in the country had average wealth (a higher wealth for both countries), then one would divide the median projected death rate further by the net worth impact factor (NWIF) for the average wealth.

## 3 Results

**Fig. 3:**
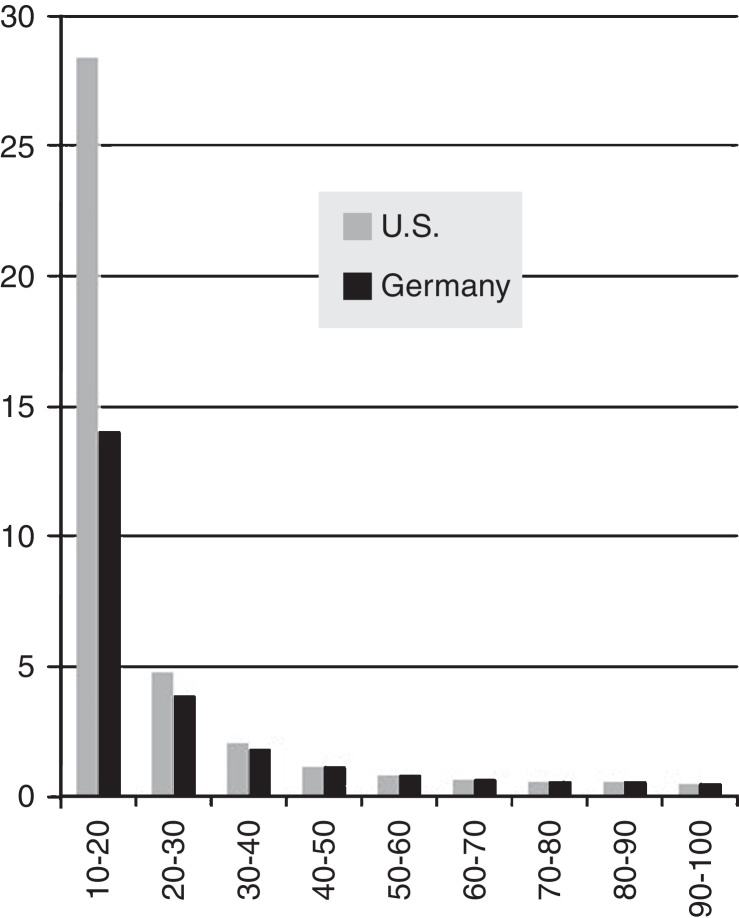
Motorway death rate inhomogeneity adjustment factors by wealth percentile.

Fig. 3 shows a strikingly higher death rate inhomogeneity adjustment factor for the U.S. data for the 10–20 percentile bin, and somewhat higher factors in the next two bins. Obscured by the scale of the chart, the German 50–60 percentile bin is slightly higher than the U.S. and others are about even. Death rates for the upper percentile brackets are so low they are unimportant in the calculation. The big factors are in the 10–30 percentile range, which does not heavily influence popular measures of “inequality” which focus on the extremely wealthy. How much difference do these factors make in motorway death rates?

The average of the adjustment factors for the U.S. is 3.96, and for Germany 2.42. One can conclude that if Germany were homogeneous at its median wealth, then its motorway death rate would be reduced to 4/2.42 = 1.65 per 100,000. Similarly, the median homogeneous projected rate for the U.S. would be 11.3/3.96 = 2.88. These are closer, but the U.S. is still 1.75 times higher.

Suppose, for the sake of discussion, the average wealth of each country were distributed approximately evenly. And further suppose that the psychology of the citizens came into a normal balance with the newfound wealth (or poverty, depending on which direction it changed for the individual), consistent with the OECD-derived 50% weighting factor for motorway deaths. Then the average wealth homogeneous motorway death rates for the two countries would be U.S. 0.75 per 100,000, and Germany 0.69. The U.S. would be in that case be only 1.09 times worse than Germany.

Keep in mind that the U.S. is safer *per mile* already. This is not a judgment on either country. Germany is much more densely populated, which might increase their rate, but has more accessible public transportation and lacks the open space that invites and requires so many miles of driving. But when all inhomogeneity factors are accounted, the U.S. and German rates per capita are closer than they have any right to be, given the uncertainty in the OECD-derived weighting factor, the differences in driving conditions, and the omission of the bottom percentile bin from both calculations. The explanatory power of economic inhomogeneity is surprising.

Notice that there is no interaction between bins assumed in our method. There is no effect postulated of poor drivers involving good drivers in accidents, nor is there an effect postulated that good drivers dilute the accident prone nature of poor drivers. In other words, the effect is not because of inhomogeneity per se. It is purely an economic effect, computed as a linear sum. This is a relatively benign case, yet it seems consistent with the data. In the case of criminal data, one does not always find that crime is associated with wealth or poverty. A uniformly poor neighborhood may in fact have a reasonably low crime rate. Instead the crime rate is sometimes found to increase with inhomogeneity itself [19].

The crash rate equation suggests a particular kind of explanation of crime due to inhomogeneity. The mixing of wealth levels provides ready accessibility of high value targets for criminals, that is, the parameter *V_f_* can be large in a mixed neighborhood. What's really important in this case is the ratio of *V_f_*/*C_c_*, where *C_c_* is essentially the cost of getting caught. Had non-fatal accidents been included, there might possibly have been such an effect, as for example when a driver induces an accident with a supposed wealthy or insured driver and feigns injury to obtain damages in a lawsuit. However, by restricting our data to fatal accidents, the motivation is removed.

What has been seen is that the data are subject to interpretations and variables, is that a quantitative risk equation, developed for entities assumed to behave purely on economic grounds such as corporations (presuming if they don't, a competitive market will eliminate them), *can* be applied to inhomogeneous populations of actors, and through weighting adjustments can even be applied where the ultimate motivations transcend economics.

It was also seen that the unequal distribution of wealth, however fair and just it might be on grounds of economic competition, has an impact on motorway death rates … and probably many other areas of life, such as political stability. What would happen if the wealth were simply redistributed? Germany does this to a greater extent than the U.S. By doing it modestly through adjustments to income, presumably the motivation of German citizens is not adversely affected. But the crash rate equation suggests that if direct grants for poverty are used, the real cost of a crash is eliminated. A citizen who loses a grant would presumably get another one. So care must be taken before jumping to conclusions about wealth redistribution. Redistribution in both the U.S. and Germany is based on income, not wealth. Our study only shows a projected correlation between wealth and motorway fatalities. Although the crash rate model assumes a causal relationship from wealth (*C_c_*) toward risk behavior (fatalities), this study cannot disprove that the relationship might be in the reverse direction, or that wealthy people might not have higher fatality rates associated with flying or boating, etc.

Similarly, if one simply make the cost of mistakes (let's assume non-fatal ones) as high and permanent as possible, then in that case also, the costs of future mistakes of the citizen so penalized are reduced dramatically, if they have little left to lose. This is not in the benefit of society either.

Both risk homeostasis and crash rate analyses are typically published in specialized journals and books only read by risk and safety practitioners. It does seem that with this cross-technique success: the possible ability to quantify risk homeostasis and apply the theories to inhomogeneous populations and social issues, it is advantageous for a wider spectrum of investigators to have awareness of the methods. Use of new methods of productivity and technology are already restrained by the necessity to screen for malicious or unintended use. Consider airport security screening as an example of industrial screening to reduce a defect ratio *D*. Collection of phone records by the U.S. National Security Agency looking for patterns of terrorism is another effort to reduce the defect ratio. A civilization capable of providing star travel would have routine operation of vehicles whose kinetic energy might dwarf a mere asteroid, and whose misuse might dwarf major extinction events in planetary history. The ability to create life with arbitrary genetic codes has similar impact, perhaps more so than one can even imagine.

## 4 Conclusion

This paper has attempted to show that two relatively recent additions to the theoretical toolbox can be combined, along with a method of addressing inhomogeneity, to give a remarkable explanation of data previously considered a puzzle. Both theories have other mechanisms for affecting risk outcomes. These results suggest that these are either applied similarly or under-utilized in U.S. and German roadway safety approaches, probably some of both.

The context of the research derives from multiple disciplines. From highway safety research, a correlation between wealth and motorway death rates is taken. From system engineering and project management, a theory of crash rate is used which relates risk, reward, and accident costs. The methodology has been to use the wealth vs. death rate data in a general way to estimate individual statistical motorway death rates via a weighting factor applied to wealth, i.e. wealthy people have more to lose. A second step in the methodology was to use 10 percentile bins to characterize the wealth of a population and compute the motorway death rate as a weighted summation, normalized so that the median wealth matches the population's actual motorway death rate. Per-capita data were used to avoid arbitrary bias from the construction of roads in little utilized areas.

Civilization should go forward and not flinch from great possibilities like travel to the stars or creation of life (either biological or robotic). At the same time, one cannot expect to go on managing risk with the sum-of-parts no-feedback tools that originated in Mesopotamian witchcraft 5000 years ago [Ibid. 1]. As a society it is desirable to explore and use all tools available.

Several topics for further investigation present themselves. In the cases of financial crises, and of civil disturbance which cause refugee migrations, people often refer to the “contagion” of crises. In the context of the crash rate model these crises amount to “crashes” of society. There are two stages of contagion. Regardless of what originates the crisis, it spreads to other members of the same society because it stresses (or sometimes destroys) the systems and institutions of the economy and social order. Second, by market linkages or migrations the crisis spreads to other societies. In our view these linkages indicate that the world's largest economy (or second largest depending on the latest Chinese data), the U.S., combined with the highest level of inhomogeneity of any developed nation, poses a significant global risk.

The reader may react that this is a contrarian view, as one is accustomed to thinking of the stressed or belligerent nations as posing the greatest threats. Consider that the U.S. as a consumer nation entices others to become dependent on its markets. The 2009 Financial Crisis originated in the U.S., and was intimately connected with inhomogeneity. Incentives to provide housing loans to those who could not otherwise afford them were abused, resulting in loans to those who could not afford them at all. In a sense, the upper percentile brackets, financial interests were exploiting the lower percentile brackets (who were in many cases willingly exploited, judgment is not intended, only to note the role of inhomogeneity).

Ukraine and Russia suffered along with many other nations from the U.S. Financial Crisis and are not recovering, contributing to their conflict, which began over proposed membership in the EU to increase employment and economic growth. Those countries do not even have a significant amount of trade with the U.S., but the countries they trade with do, and so the crisis spread by contagion. As is widely known, the U.S. recovered gradually over the next half dozen years, but many poorer nations including Ukraine, Tunisia, Syria, Yemen and others that are now trouble spots did not recover. The Tunisian man who immolated himself to start the Arab Spring shouted no slogan against the U.S., nor any religious slogan, but instead said, “I just want to work.” And how is Tunisia's economy doing? Unemployment is currently 15.3%, down a bit from 2011 but up from pre-revolution 13% [20]. The spike upward began in mid-2010, at which time outward migration also began growing at over 6%, with most headed to Europe [21]. That *increases* in inhomogeneity (inequality) reduce long term economic growth and lead to limited labor mobility, erosion of self-esteem, unrest and conflict is established in the literature [22]. Noted economist Robert Shiller cites rising inequality, especially in the U.S., as the “most important problem” [23].

Wealth inhomogeneity is a subject of active discussion and investigation in the U.S. Since 1979 after tax income of the top 20% has grown at double the rate of the middle three quintiles. The bottom quintile has done slightly better [24]. Wealth inhomogeneity is much greater than income inhomogeneity. Analysis reveals that lower deciles catch up (in percentage terms) in wealth during their lifetimes, due to the influence of their salaries. Almost none of the “catch up” is passed on to the next generation, possibly due to cultural emphasis on financial planning only for retirement, whereas the top quintile starts out with some of the previous generation's wealth [25].

With regard to trends for wealth (or income) inhomogeneity worldwide, two important areas are trade and productivity growth. Reliable Gini indexes are hard to obtain from some important trading nations. Despite a plan to eliminate “extreme poverty” by 2022, China's Gini index has been growing and in 2000 when China's reached .412 (.4 or greater is considered severe), their National Bureau of Statistics (NBS) stopped releasing data. When a university released a figure of .61 in 2012, NBS starting releasing data again. The 2012 number is .474, slightly better than the U.S. coefficient of .477 [26]. Productivity growth has been extensively researched for its role in forcing increases in money supply to avoid unemployment, financial crises and deflation [27]. A recent study considering new automation factors of big data, pattern recognition, and robots with advanced sensors and dexterity suggests within two decades as much as 47% of existing jobs could be automated [28]. Increased technology, associated with productivity growth, makes ever greater skills a requirement for high paying jobs [29], requiring more investment by employees in education, leading to student debt, increasing inequality. Stiglitz suggests market competition should promote businesses to cut prices, profits and large compensation for high end workers and executives [30], thus reducing inequality, but central bankers are not cash-limited rational market participants [Ibid. 21], and act to ensure restoration of employment after a downturn, not the restoration of wealth lost due to re-training and the buildup of working class debt.

Thus increases in inhomogeneity may not be the inevitable result of capitalism as Marx supposed [31], but unintended consequences of monetary policy. There might be some correlation with John Galbraith's observation of greater inequality in countries with large financial sectors [32]. As the U.S. Federal Reserve officially targets both unemployment and currency stability, and the EU central bank primarily the latter, it may be productive to examine this difference further for a possible unintended role due to the unemployment target feedback control in shifting assets from workers for which it is a leading indicator (when it rises, the potential to accumulate wealth begins), to investors and business for which it could be interpreted as a lagging indicator (when it rises, capital has already been made available for hiring). If confirmed, mitigation strategies could be found which are not confined to the old ideologies of the left or the right, or strategies doomed to be marginalized by accelerating productivity and technology, and which may at least remove unnatural inhomogeneity caused by the unintended effects of economic stability mechanisms.

## Declarations

### Author contribution statement

Robert L. Shuler: Conceived and designed the experiments; Performed the experiments; Analyzed and interpreted the data; Wrote the paper.

### Funding statement

The author received no funding from an external source.

### Competing interest statement

The author declares no conflict of interest.

### Additional information

No additional information is available for this paper.
